# Electromagnetic Fields for the Regulation of Neural Stem Cells

**DOI:** 10.1155/2017/9898439

**Published:** 2017-08-28

**Authors:** Mengchu Cui, Hongfei Ge, Hengli Zhao, Yongjie Zou, Yujie Chen, Hua Feng

**Affiliations:** Department of Neurosurgery, Southwest Hospital, Third Military Medical University, Chongqing, China

## Abstract

Localized magnetic fields (MFs) could easily penetrate the scalp, skull, and meninges, thus inducing an electrical current in both the central and peripheral nervous systems, which is primarily used in transcranial magnetic stimulation (TMS) for inducing specific effects on different regions or cells that play roles in various brain activities. Studies of repetitive transcranial magnetic stimulation (rTMS) have led to novel attractive therapeutic approaches. Neural stem cells (NSCs) in adult human brain are able to self-renew and possess multidifferential ability to maintain homeostasis and repair damage after acute central nervous system. In the present review, we summarized the electrical activity of NSCs and the fundamental mechanism of electromagnetic fields and their effects on regulating NSC proliferation, differentiation, migration, and maturation. Although it was authorized for the rTMS use in resistant depression patients by US FDA, there are still unveiling mechanism and limitations for rTMS in clinical applications of acute central nervous system injury, especially on NSC regulation as a rehabilitation strategy. More in-depth studies should be performed to provide detailed parameters and mechanisms of rTMS in further studies, making it a powerful tool to treat people who are surviving with acute central nervous system injuries.

## 1. Introduction

In 1985, Barker et al. demonstrated the possibility of noninvasively influencing both the central and peripheral nervous systems via localized magnetic fields (MFs) that could easily penetrate the scalp, skull, and meninges, thus inducing an electrical current in the brain or peripheral nervous system [1]. Now, this technique is primarily used in transcranial magnetic stimulation (TMS), which can be administered in different forms and appears to induce specific effects on different regions or cells that play roles in various brain activities. Studies of repetitive transcranial magnetic stimulation (rTMS) have led to novel and attractive therapeutic approaches [2, 3]. Different rTMS techniques, such as single, paired, or repetitive trains of intermittent theta burst stimulation (iTBS), have been commonly applied to many refractory neuropathy conditions, such as degenerative diseases, malignant tumors, and traumatic diseases, and especially in neurology and psychiatry for both diagnostic and therapeutic purposes [4]. Interestingly, the various means of stimulation exert completely different regulatory effects because high-frequency rTMS (defined as >5 Hz) stimulates enhanced cortical excitability and produces long-term potentiation (LTP). In contrast, low-frequency rTMS (defined as <1 Hz) decreases cortical excitability and induces long-term depression (LTD) [5]. However, the intrinsic cellular and molecular mechanisms underlying rTMS(MF)-based therapies are still elusive.

Neural stem cells (NSCs) are a type of self-renewing stem cell which possess the multidifferential ability to produce neurons and glia in the nervous system during the embryonic period. Some NSCs persist in the adult mature brain, and their capacity to differentiate into multiple cell types allows them to produce neurons throughout the lifespan [6]. They maintain homeostasis and repair damage [7]. Compared to differentiated cells, adult stem cells can proliferate to sustain themselves and differentiate into one or more specialized cell types within a certain cell lineage [8]. As such, for the ultimate purpose to regenerate and recover normal functions, stem cells are a promising tool for tissue or organ repair. In particular, adult stem cells are most often in a quiescent state and can be triggered by intrinsic or extrinsic factors or their complicated combination to initiate self-renewal and differentiation [9, 10]. The current consensus is that a series of niche signals and cellular intrinsic processes are involved, and several researchers have made great efforts to identify the roles of these signals and processes in brain physiology and explore their potential use in cell-based therapies to treat neurological or neurodegenerative diseases [11, 12]. In the adult mammalian brain, the subgranular zone of the hippocampal dentate gyrus and the subventricular zone of the lateral ventricles are two of the main areas where NSCs reside [13, 14]. The production of new neurons maintains the NSC pool [15], and thus, NSCs or neural progenitor cells (NPCs) are the most important component in brain regeneration and plasticity. The nervous system is perhaps the most difficult system to repair in regenerative medicine. In addition, adult NSCs are located deep in the body and are few in number. Although SCs have been studied for decades, how to influence these cells noninvasively and efficiently for specific applications remains a challenge. The latest research has reported that rTMS(MF) has a variety of effects on adult NSCs, shedding light on possible cures for intractable diseases, cerebral trauma, stroke, depression, dementia, Parkinson's disease, and so forth Therefore, strategies to generate a MF to stimulate endogenous processes of NSCs have gained considerable interest, but the behavior of NSCs in the context of rTMS(MF) therapy needs further elucidation [16]. Focused on the integration of two promising approaches, MFs and NSCs, this review will discuss the effects of MFs on NSCs and the potential mechanisms as well as provide an outlook regarding future directions. Thus, noninvasive MF stimulation, specifically transcranial magnetic stimulation (TMS) on NSCs, may be a promising option.

## 2. The Electrical Activity of Neural Stem Cells

**Figure 1:**
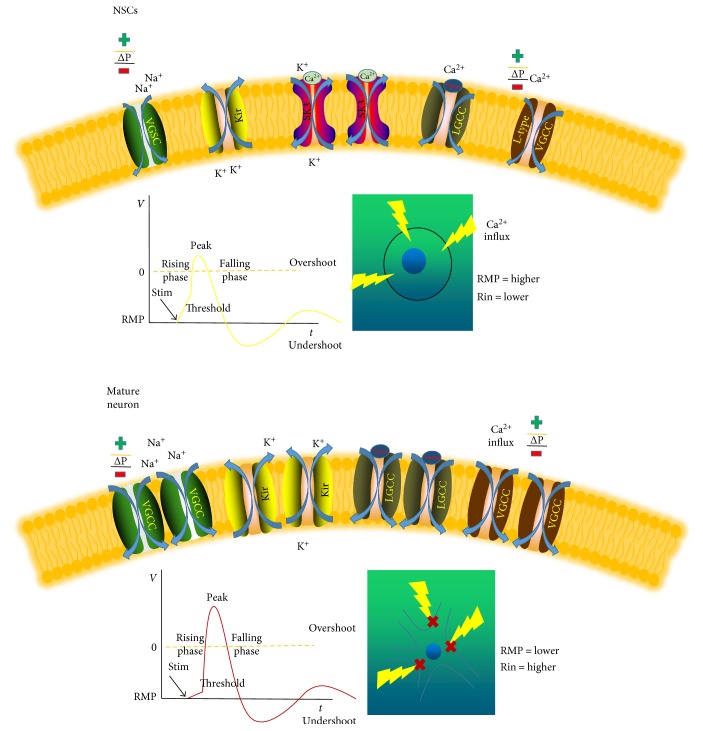
The electrophysiological differences between NSCs and neurons. Compared to mature functional neurons, NSCs exhibit a higher RMP and a lower Rin due to shortage of Kir; moreover, it is not easy for NSCs to generate AP because of a lack of VGSCs. VGCCs, mostly functional L-type and LGCC, are probably the main roles in modulating intracellular Ca^2+^ concentration especially L-type VGCCs, Ca^2+^-dependent K^+^ channel SK3 abundantly expressed in NSCs is responsible for the migration and proliferation.

NSCs are a type of immature, undifferentiated cells. Due to their property of “stemness,” their physiological features, especially electrophysiological characteristics, are distinct from well-known neurons, and they realize their functions mainly based on electrical activities. It is widely known that neural cells have specific excitability and that ion channels are the molecular foundation enabling them to generate electrical activities. Neural cells and NSCs participate in intercellular signaling transitions and transmembrane signaling transduction, which are the prerequisites for cells to become physically activated to exert their functions (Figure 1). During the course of NSC differentiation, the expression of ion channels and their states are continually varied to accommodate the microenvironment (niche) of different periods. Although studies of NSCs have become gradually more in-depth, the characteristics of NSCs cultured and differentiated *in vitro* have been primarily explored based on morphology and immunocytochemistry, whereas few studies have performed functional identification of NSCs. Studies indicate that neural cells under different conditions are characterized by different electrophysiological features, and the development and differentiation of ion channels are precise indicators of specialized NSCs. Their unique electrophysiological properties not only provide a new and efficient functional means to better identify NSCs and types of differentiated neural cells but also help to elucidate the mechanisms underlying MF stimulation and NSCs.

### 2.1. Passive Membrane Properties of NSCs

Resting membrane potential (RMP), membrane input resistance (Rin), and membrane capacitance (Cm) are major parameters of the passive membrane properties of cells. Liu et al. [17] reported that epidermal growth factor/basic fibroblast growth factor- (EGF/bFGF-) reactive neural progenitor cells originate from the subventricular zone (SVZ) or spinal cord of rats and can be classified into three types based on their *I*-*V* curve: type I exhibits delayed outward currents, type II exhibits no rectification, and type III exhibits outward and inward rectifying currents. Significant differences in the passive parameters have been found among the three types of cells. Compared with other types, type I cells are characterized by a high Rin and a low RMP. Liu hypothesized that immature type II neurons may be glial cells, while type III cells may be undifferentiated NSCs. This hypothesis corresponds with the work of Doetsch et al. [18] on the morphology of NSCs. The major transmembrane channels of glial cells are dense passive K^+^ channels, which result in a higher RMP and a lower Rin. As NPC differentiate, RMP increases while Rin decreases due to the addition of passive K^+^ channels, but they are still distinct from mature neurons, reflecting immature differentiation. By studying hippocampal slices of nestin promoter-GFP transgenic mice to directly observe fluorescent cells, Fukuda et al. [19] classified neurons into two species according to the levels of Rin and RMP: type I with low Rin and high RMP and type II with high Rin and low RMP. In addition, Fukuda et al. performed a classical morphological identification and found that type I cells are GFAP-positive and polysialic acid neural cell adhesion molecule- (PSA-NCAM-) negative, while type II cells are GFAP-negative and PSA-NCAM-positive, in agreement with the reported functional outcomes of NSCs at different stages.

Certainly, NSCs/NPCs can be distinguished from each other during different periods of life. Compared to adult NSCs, neonatal and embryonic NPCs exhibit a more depolarized RMP between −55 mV and −40 mV [20–24]. The Rin of neonatal NPCs is different under different circumstances; for connected cells, the Rin is 150 MW, whereas for isolated cells, it is 650 MW in the presence of a gap junction blocker [20]. In addition, *in vitro*, the Rin of embryonic NPCs is 1 GW [23]. Thus, adult NSCs/NPCs have a more hyperpolarized RMP and a lower Rin than embryonic and neonatal NSCs/NPCs. This disparity may indicate the morphological and functional changes that the neonatal or embryonic SVZ undergoes during developmental shifts in the neurogenic niche that are distinct from the adult SVZ. Above all, these studies indicate that different types of neural cells have different electrophysiological features of their passive membrane.

### 2.2. Ability to Generate the Action Potentials (APs)

Neural cells transmit excitatory signals via APs, but the ability of NPCs to generate APs significantly differs across stages of life. Neurons can be evaluated based on their action potential duration (APD) 50 and APD 90, namely, the time required for 50% and 90% repolarization of the AP, respectively. A shortage of Na^+^ channels makes it difficult to induce Aps; however, as the expression of ion channels increases, the *I*-*V* curve begins to show a rectified performance, and transmembrane ionic currents prompt the opening of more channels, resulting in positive feedback. Compared with mature neurons, the majority of studies have shown that there is no spontaneous AP in NSCs, and APs are induced by depolarization of distant mature neurons. Liu et al. [17] found that further differentiated cells hold a greater ability to generate APs, longer duration APs and higher amplitude APs, which are related to a lack of voltage-gated Na^+^ channels (VGSCs), particularly tetrodotoxin- (TTX-) sensitive Na^+^ channels. In the process of specialization, as the expression of Na^+^ channels increases, the amplitude of APs is augmented. Therefore, the development of Na^+^ channels is closely related to shortened durations of APs, indicating that more Na^+^ channels are involved for the same level of depolarization stimuli.

### 2.3. Voltage-Gated Ion Channels of NSCs

There are four major types of voltage-gated ion channels: Ca^2+^, Na^+^, K^+^, and Cl^−^ channels. Some of these channels are opened through membrane depolarization, while some are opened through membrane hyperpolarization. In terms of their voltage sensitivity, researchers most often focus on excitable cells. In neurons, these channels play a key role in neuronal functions, such as in synaptic transmission, the generation of APs, membrane current transmission, long-term potentiation, and the modulation of gene expression. However, the role of VGSCs in NSCs has been ignored because of their inexcitability. It is well known that glial cells are the insulation of the central nervous system and also exhibit VGSC currents. However, the quantity or quality of VGSC currents in glial cells are too small to generate APs, and their functional connectivity is controversial [25]. Furthermore, as an immature cell type, adult NSCs are unable to produce APs in response to depolarizing currents [26, 27]. Similarly, embryonic and neonatal NSCs exhibit neither VGSC currents nor APs upon depolarizing current injection [20, 24, 28, 29]. Interestingly, some (13–55%) cultured NPCs have small transient inward VGSC currents [23, 26, 27, 30]. Given that experiments carried out *in vitro* may not translate to *in vivo* situations, the expression of VGSCs in NSCs/NPCs required confirmation by electrophysiological studies in situ. K^+^ channels, whose gene expressed before differentiation corresponds to both voltage- and Ca^2+^-dependent types, are widely found throughout the brain, regardless of the cell type (neurons or glial cells). K^+^ currents can be probed both before and after differentiation. In addition to their primary role in the routine modulation of neuronal excitability, K^+^ channels also generally participate in the regulation of critical processes such as membrane potential, proliferation, and apoptosis across a wide range of cellular activities [31–34]. K^+^ channel currents are usually recognized as two classes: inward and outward K^+^ currents. Outward currents include rapidly activated and inactivated transient currents such as A-type K^+^ (KA) channel currents, which are highly sensitive to 4-aminopyridine, and slowly inactivated or non-inactivated currents such as delayed rectifying K^+^ (KDR) channel currents, which are rarely inactivated and are tetraethylammonium- (TEA-) sensitive due to their unique properties. Inward currents include inwardly rectifying potassium channel (Kir) currents that are mainly responsible for the RMP. KDR channels play a critical role in repolarization and are rarely found in immature cells. Wang et al. probed KDR and Ca^2+^-dependent K^+^ channels in mouse brain slices 15~25 days after birth. Liebau et al. [35] found hyperexpression of a class of Ca^2+^-dependent K^+^ channel (SK3), which plays a variety of roles in many physiological processes of NSCs.

Voltage-gated Ca^2+^ channels (VGCCs) comprise three major subfamilies: CaV1.x, CaV2.x, and CaV3.x. They are extensively expressed in neurons and are responsible for modulating gene expression, neurotransmission, and various fundamental Ca^2+^-dependent intracellular events such as differentiation, apoptosis, and proliferation [26, 27, 34]. VGCCs are considered to play functional roles in early neuronal development [36]. In adult NPCs, however, the functional modulation and roles of VGCCs are unclear. Most recent studies have investigated L-type (CaV1.2) and T-type (CaV3.1) VGCCs in adult cultured NSCs and have detected their expression at the transcriptional or translational levels [37]. *In vivo*, ischemia-induced neurogenesis in adult mice can be blocked using L-type VGCC antagonists, but there is no evidence to show that they exert an effect on basal neurogenesis, suggesting a special role of VGCCs in disease-related neurogenesis [38]. Ca^2+^ transients are rarely found in the majority of NSCs, whereas small VGCC currents (Ca^2+^ transients) can be detected using a higher depolarization level induced by high K+ concentration (100 mM) in adult NPCs [37]. Furthermore, no reports have detected a significant VGCC inward current in NPCs using physiological recording methods [27]. However, considering that large outward K^+^ currents may mask small Ca^2+^ currents, conditions should be changed to detect such small currents. Previous studies of changes in the concentration of intracellular Ca^2+^ caused by activation of Ca^2+^ channels in NSCs are limited despite the importance of Ca^2+^ in migration, proliferation, and differentiation. One can question the functional role of masked small VGCC currents and the mechanism of membrane depolarization that activates VGCCs. In the future, it is important to determine the clear mechanism by which intracellular Ca^2+^ concentrations can effect cellular activities, particularly those that affect NSC function. In addition to VGCCs, NSCs also express another type of channel at the transcript level. The canonical transient receptor potential channel 1 (TRPC1) channel, a voltage-gated Ca^2+^ channel [37], plays a key role in Ca^2+^ influx and proliferation of NSCs [39]. The above findings may suggest that Ca^2+^ may be the key factor in studying the mechanisms of NSC functional activities. In addition, even the outcomes among studies may differ, and interfering factors such as animal species, tissue origin, tissue parts, immediately extracted NSCs versus cultured NSCs, and single-cell recordings versus brain slice recordings should be noted. In general, it is widely accepted that undifferentiated NSCs exhibit a high RMP, a low Rin, and inward-rectifying potassium currents without inward Na^+^ currents.

## 3. The Fundamental Mechanism of Electromagnetic Fields

Noninvasive transcranial magnetic stimulation (rTMS) produces an electromagnetic field that can easily penetrate the skin and skull to influence the brain with little decay [40]. Electromagnetic fields act on the brain and induce currents based on the Faraday electromagnetic effect. Repeated electromagnetic fields can also affect the refractory period and influence connective horizontal neurons to modulate the balance between excitation and inhibition. In addition, electromagnetic fields induce electric currents whose function in the brain to change the excitability of cells depends on the intensity and frequency of the stimulation. It is currently accepted that low-frequency rTMS (defined as <1 Hz) diminishes the excitability of neuronal cells, whereas high-frequency rTMS (defined as >5 Hz) enhances neuronal excitability, resulting in the modulation of brain activity [41, 42]. Solid evidences from clinical tests such as positron emission tomography (PET) [43] and functional magnetic resonance imaging (fMRI) [41] also support this fact. Different frequencies of stimulation may exert different effects on brain metabolism; high frequency may increase the metabolism level, while low frequency may decrease the metabolism level and cerebral flow. Furthermore, electromagnetic fields can regulate neurotransmitters both at the transcript level and expression level, which may provide an alternative route to help elucidate potential mechanisms. In studying the activities of cells involved in signal transduction, we should consider physical mechanisms combined with transduction, and ion channels may be the fundamental factors that are initially modulated.

## 4. Effects of Electromagnetic Fields on Neural Stem Cells

A general survey of the present study shows that a series of different parameters, including intensity, frequency, orientation, and distance, and models, have been extensively applied and studied. Many of these studies are specific to the model; therefore, rTMS and NSC studies are complicated and difficult to classify. Fortunately, the number of studies in the field is small, the details of which are reported above.

Francis et al. found that the exposure of adult mice to ELFEFs *in vivo* produces a significant enhancement in the number of newborn neurons in the GCL of the DG [44–46]. The vast majority of those that survive differentiate into immature neurons and then mature granule cells, which migrate into the GCL. The expression of NeuN (commonly considered a marker of differentiated neurons) was investigated 4 weeks after protocol. Compared with estimates based on DCX labeling right after exposure, the total number of newly generated neurons was markedly reduced. In exposed and control mice, less than half (45% and 48%, resp.) of the newly generated immature neurons (DCX+) had become mature NeuN-expressing cells. These observations are consistent with previous reports showing that later-born granule cells localize predominantly in the inner core of the GCL [47].

The BrdU and nestin-corporation method shows us that EMFs can also increase the number of BrdU and nestin-positive cells within the area between the SVZ and lesion at 7 and 14 days after lesioning, indicating that EMF exerts a positive effect on the proliferation and migration of NSCs [48]. Cuccurazzu et al. [49] showed that extremely low-frequency and low-intensity EMF stimulation promotes adult hippocampal neurogenesis. In addition, Arias-Carrion et al. [50] showed that transcranial magnetic field stimulation promoted neurogenesis by the SVZ cells in nigrostriatal lesions.

Abbasnia et al. [16] found that with both low (1 Hz) and high (30 Hz) frequency rTMS, there is a marked rise in the proliferation of NSCs in the adult murine intact brain 2 weeks after application. An increase in the frequency of neurosphere formation and the size of the neurosphere was also observed in the rTMS-treated animals throughout the experiment. Furthermore, differentiation of the induced neurospheres showed that both NSCs treated with either the one-week or two-week rTMS protocol were more neurogenic than those of the sham-treated group. Moreover, *in vitro*, both 1 Hz and 30 Hz rTMS treatments applied for one week promoted NSC proliferation and neuronal differentiation. Interestingly, their findings also showed that there is no difference between low-frequency rTMS and high-frequency rTMS in terms of promoting NSC proliferation and increasing their neurogenesis. However, a marked increase in the quantity and size of neurospheres was observed for one week following both low- and high-frequency rTMS, indicating that only the discrepancy in neurosphere size (diameter) with low-frequency rTMS reached statistical significance. This implies that even one week of low-frequency rTMS stimulation results in a subtle increase in NSC proliferation. To understand these findings, the authors prolonged the application time (2 weeks) of the low- and high-frequency rTMS application. Given that low-frequency rTMS and high-frequency rTMS are similarly effective, they concluded that compared to high-frequency rTMS, low-frequency rTMS may be a safer and more tolerable therapeutic option with fewer risks [16]. In addition, intermittent theta burst stimulation (iTBS), which is a newly developed rTMS therapeutic protocol, was studied by Luo et al. [51], who compared it to the conventional 20 Hz high-frequency rTMS in an ischemic model. Their results showed iTBS significantly enhanced NSC migration and differentiation in the peri-infarct striatum, indicating that differences among different parameters may exist, and further studies are needed to clarify the effects of rTMS on NSCs.

However, experiments investigating only one parameter while controlling for all other parameters have drawn some useful conclusions. Studies focusing on the effects of high-intensity pulsed electromagnetic stimulation (HIPEMS) on the proliferation and differentiation of neonatal rat NSCs *in vitro* were carried out by Meng et al. [52]. NSCs isolated from neonatal rats were exposed to HIPEMF (0.1 Hz, 0.5–10 Tesla (T), 5 stimuli). A control group was correspondingly included. Given that a high number of stimulations (>30) might exert a suppressive effect on the growth of NSCs, Meng set the stimulus number to a low value—5 times per experiment. After a series of protocols were performed, they found that with 5 0.1 Hz frequency stimulations, rat NSCs showed poor *in vitro* growth in the HIPEMF 6.0–10.0 T peak intensity group, whereas a significant enhancement in the proliferation of rat NSCs was observed in the 0.5–4.0 T peak intensity, HIPEMF-stimulated group. The results showed that NSC proliferation in the 3.0 T and 4.0 T HIPEMS groups were remarkably higher than that of the other groups after 24 to 168 h of stimulation. Therefore, no linear relationship exists between the groups in terms of the proliferation of NSCs; the 6.0 T, 8.0 T, and 10.0 T groups were lower than the control group, indicating that high-intensity stimulation restricts the growth of NSCs. Flow cytometry was applied to detected the rate of neuron-specific enolase-positive neurons, and the results showed there were no differences between the HPEMS groups and the control group. Therefore, we can conclude that HIPEMF promotes the proliferation of rat NSCs *in vitro* under a certain range of intensities and fixed parameters. Furthermore, there is a window effect, with 4.0 as the critical value, suggesting a linear strength-effect relationship within the peak intensity range of 0.5–4.0 T in promoting the proliferation of NSCs.

There are few related studies of the effects of rTMS on NSCs, few of which have investigated the effects of proliferation and differentiation *in vivo* [53–55]. Ueyama et al. [53] employed a BrdU-labeling method to investigate the effect of high-frequency (25 Hz) rTMS (1000 pulses/day) on neurogenesis after 14 days of application. The results showed increased cell proliferation in the dentate gyrus of the hippocampus, with most cells expressing the neuronal marker. A similar study was carried out by Feng et al. [54] in a chronic rodent model of depression. The author applied high-frequency (15 Hz) rTMS (1000 pulses/day) for a period of approximately 21 days and found an incremental increase in hippocampus cell proliferation, indicating increased neurogenesis. In a rat model of focal cerebral ischemia, 7 days after the application of high-frequency (10 Hz) rTMS (300 pulses/day), Guo et al. [55] observed a significant increase in the proliferation of NSCs in the SVZ of the lateral wall of lateral ventricle.

Although several studies of the effect of TMS(MF) on NSCs have been performed, there is no systematic analysis of MFs and NSCs due to the complexity of NSCs or to the large scale of the MF parameter. Nevertheless, according to the present study, we can conclude that rTMS(MF) is able to promote proliferation, differentiation, migration and inhibit apoptosis of NSCs in a conventional way. We also show that different strengths and different numbers of stimuli can induce different effects of HIPEMF on NSCs, indicating that there exist several potential routes for further exploration.

## 5. Potential Mechanisms of Electromagnetic Field Regulation on NSCs

Possible mechanisms behind the effects of rTMS NP/SCs have not yet been very well characterized. A thorough understanding of the underlying mechanism may help to optimize the stimulation protocol, characterize how EMF exerts its effect in animal models at the molecular level, and increase the translation of results to humans, thereby increasing their application in the clinic and proving an effective tool for clinicians.

### 5.1. High-Frequency rTMS Enhances the Expression of BDNF

Several studies have reported that BDNF is a key factor for increased hippocampal cell proliferation and neuronal differentiation after the application of rTMS [54]. In addition, reports show that in several brain areas of rats, including the hippocampal CA1 and CA3 subfields, high-frequency rTMS (20 Hz) stimulates the expression of BDNF [56]. In addition to the increase in BDNF expression, the expression of pERK1/2 was also increased [57], indicating rTMS might activate the BDNF/ERK signaling pathway to upregulate cell proliferation in the hippocampus.

### 5.2. The miRNA-106b-25 Cluster in a Model of MCAO Stimulated by High-Frequency rTMS

A number of miRNAs play a role in the determination of NSC fate, including NSC differentiation and proliferation [58–60]. Given the significant effects that rTMS exerts on gene expression, it is possible that rTMS also has the potential to modulate miRNAs. Guo et al. found that 10 Hz rTMS stimulation in a rat model of cerebral ischemia resulted in a remarkable enhancement of miR-25. Brett et al. demonstrated that the miRNA-106b-25 cluster could also promote the proliferation of adult NSCs [61, 62]. However, there was a significant decrease in its corresponding factor-target gene p57 [63, 64]. As we previously illustrated, p57, which can be suppressed by mir-25, is a Cdk inhibitor (CKI) that binds to Cdks to modulate transitions between cell cycle phases. Proteins of the Cip/Kip family inhibit the transition from G1 to S, thereby regulating the cell cycle; therefore, they proposed that rTMS might increase the expression of miR-25 in order to repress its target gene p57, thereby, as mentioned above, promoting adult NSC proliferation and inhibiting cell-cycle arrest. Moreover, the researchers also found that when miR-25 is inhibited, the proliferation of NSCs located in SVZ was also blocked. In summary, rTMS mainly activates the miR-25/p57 signaling pathway, which is responsible for the enhancement of adult NSC proliferation after focal cerebral ischemia. However, Liu et al. [65] performed a corresponding experiment for miR-106b and demonstrated that in rats with focal cerebral ischemia, the miR-106b-25 cluster increased NSC proliferation *in vitro* after high-frequency rTMS, the effects of which were dose-dependent [6]. They also showed that the miR-106b/p21/Cdk/cyclin pathway plays as an important role in this process. Interestingly, they also found that the trend for miR-25 after rTMS *in vitro* is completely different compared with those for miR-106b and miR-93. As such, the results of Liu dramatically disagree with Guo's. Taken together, miR-25 may have a more elaborate and complex role in the proliferation of NSCs after rTMS, despite the discrepancy between the two experiments. However, further studies are required to determine how miR-25 is affected after rTMS in NSCs.

### 5.3. Epigenetics May Be the Central Mechanism of ELFMF

More and more proof suggests that epigenetic mechanisms, particularly chromatin modifications, may act as critical modulators of differentiation and proliferation in NSCs [66, 67]. Leone et al. [68] demonstrated a marked increase in the expression of the proproliferative gene Hes-1 as well as the neuronal determination genes NeuroD1 and Neurogenin1. Several studies have illustrated that Hes1 is a repressive bHLH transcriptional factor that prolongs the stemness of NSCs by repressing proneural gene expression [69]. In contrast, inactivation of Hes1 weakens the repression of proneural genes and correspondingly upregulates the expression of proneural genes (as Mash1, Neurogenin1, and NeuroD1), resulting in acceleration of neuronal differentiation [70–72]. Furthermore, *in vitro* studies have also demonstrated that Hes1 is a switch for NSC proliferation and neuronal differentiation. These results are consistent with the results of previous studies. Interestingly, Hes1 can also repress its own expression by binding to its promoter, leading to the disappearance of Hes1 mRNA and protein. This negative feedback mechanism may mediate the switch between differentiation and proliferation. However, before the initiation of these events, there is an initial increase in the acetylation of H3K9 and binding of the phosphorylated transcription factor cAMP response element-binding protein (CREB) on the regulatory sequence of these genes. In addition, electromagnetic field-dependent epigenetic modifications can be inhibited by the Cav1 channel blocker nifedipine, which also involved increased occupancy of CREB-binding protein (CBP) to the same locus. Leone et al. also found that NSCs isolated from the hippocampus *in vitro* and exposed to ELFEFs showed enhanced proliferation and neuronal fate specification through changes in pCREB levels at specific bHLH neuronal gene promoters and Cav1 channel-dependent modulation of H3K9 acetylation. CBP, a histone acetyltransferase that is recruited by pCREB, is therefore involved in the epigenetic changes. Furthermore, similar results were observed in *in vivo* studies. Piacentini et al. demonstrated that ELFEF applied to cortical NSCs could enhance the quantity and function of voltage-gated Ca^2+^ channels, resulting in an increase in the concentration of intracellular Ca^2+^, and Ca^2+^-mediated signaling generated by Cav1 channels plays an important role in several fundamental cellular functions including the proliferation and differentiation of NSCs [73–76]. The potential mechanisms by which Ca^2+^ signaling regulates the transcription of numerous genes include bHLH transcriptional factors and the activation of CREB [77–80]. CREB, as a Ca^2+^-dependent transcription factor, modulates the initiation of transcriptional programs, thereby exerting an important influence on adult neurogenesis [81, 82]. Furthermore, exposure to ELFEFs also leads to the accumulation of Cav1-dependent CREB phosphorylation at Ser133 in differentiating NSCs. The significance of the phosphorylation of CREB includes effectively promoting the expression of neuronal genes (NeuroD1 and Neurogenin1) and recruiting the histone acetyltransferase CBP, which can be prevented using the Cav1 channel blocker, nifedipine.

To prove this function of histone acetylation and to illustrate how CREB acts as a recruiter of histone acetyltransferases, Leone et al. [68] exposed differentiating NSCs to ELFEF and found increased H3K9 acetylation and pCREB binding to the promoters of proneuronal genes; these events could be significantly inhibited by nifedipine, thereby significantly enhancing the mRNA expression of Cav-1-dependent proneuronal genes. H3K9 is an important type of histone acetylation that loosens the compact structure of chromatin, thereby promoting the binding of regulatory sequences and increasing transcription. CBP cooperates with CREB in several molecular pathways [83], particularly those that regulate embryonic neural differentiation in the central nervous system [84]. Chatterjee et al. [85] recently showed that a CBP activator could enhance neurogenesis in adult mice. Therefore, epigenetic chromatin modifications at specific neuronal gene regulatory sequences may mediate the effect of ELFEFs on adult hippocampal neurogenesis *in vivo*.

### 5.4. Neurotransmitter Distribution Could Also Be Involved

Alternatively, another contributing factor modulating the proliferation of NSCs in the SVZ could be the variety of neurotransmitters released by axon terminals innervating that region [86]. To our knowledge, several studies have found that nerve endings are intensively distributed in the SVZ, originating either from the local neural circuitry such as GABAergic neurons of the adjacent striatum [87–89] or from distant brain regions such as dopaminergic neurons of the substantia nigra and ventral tegmental area [90, 91], and serotonergic neurons of the raphe nuclei [92]. Importantly, it is well known that GABA is an inhibitory neurotransmitter but it could also preserve the balance in proliferation and regulate the biological states of NSCs in the SVZ [88]. Moreover, dopamine [57, 93] and serotonin [92] have been shown to have a positive influence on NSC proliferation in the SVZ. Therefore, several neurotransmitter systems could be activated by rTMS to modulate the niche of NSCs in the SVZ (or other region with NSCs) to cause an increase in cell proliferation after rTMS treatment. In terms of the previous *in vitro* studies mentioned above, showing that both low- and high-frequency rTMS increase cell proliferation and neuronal differentiation, these findings suggest that electromagnetic fields in the human body itself could be a potential mechanism by which the body regulates cell proliferation and differentiation [94]. In support of this perspective and to further illustrate the mechanisms, findings at the molecular and cellular level are discussed.

### 5.5. Ca^2+^ Ion Channels Are Proposed as a Link between These Mechanisms

However, based on the present studies and the electrophysiology features of NSCs, some conclusions can be drawn: Ca^2+^ and CREB might be the hinge of effects because of a lack of excitability of NSCs. According to the Faraday effects, a possible mechanism could be that MF facilitates the intracellular and extracellular exchange of ions through long-term opened ion channels and upregulates the expression of voltage-gated Ca^2+^ channels (VGCCs) or TRPC1 channels could result in a current and potential difference of NSCs due to Ca^2+^ that floods from the extracellular matrix or endoplasmic reticulum through voltage-dependent channels or the force of MF itself. On one hand, intracellular Ca^2+^ stimulates phosphorylation of the transcription factor CREB, activating the CREB signaling pathway, and pCREB recruits more CBP to initiate the transcriptional machinery, including histone acetyltransferases. At the same time, histone modifications secondary to electromagnetic field-activated signals, particularly calcium, lead to chromatin unravelling, thereby promoting the binding of pCREB to the promoter region. On the other hand, pCREB is able to bind to the promoter of a series of miRNA to modulate their expression. In addition, miRNA as well as epigenetic mechanisms could affect the expression of BDNF, which plays a critical role in the activities of NSCs.

**Figure 2:**
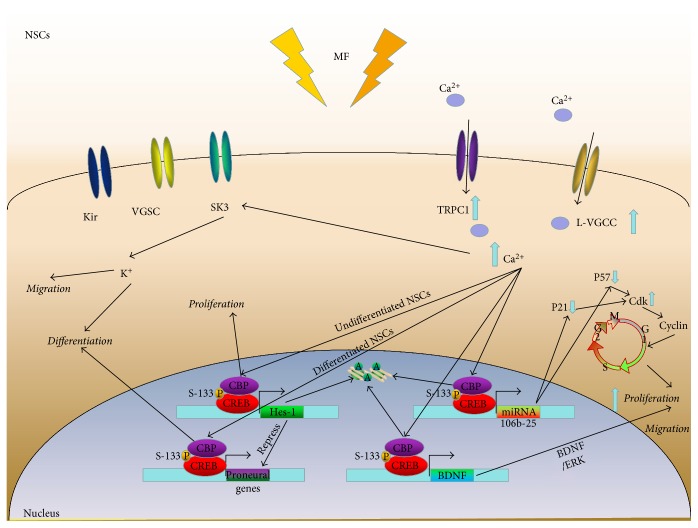
Potential mechanisms of electromagnetic field regulation on neural stem cells. Ca^2+^ and CREB might be the hinge of effects, because of a lack of excitability of NSCs, and according to the Faraday effects, a possible mechanism could be that the MFs facilitate the exchange of intracellular and extracellular ions through these long-term opened ion channels and upregulate the expression of voltage-gated Ca^2+^ channels (VGCCs) or TRPC1 result in a current and potential difference of NSCs, Ca^2+^ flood into from extracellular matrix or endoplasmic reticulum through the voltage-dependent channel or the force of MF itself; on the one hand, intracellular Ca^2^^+^ stimulates phosphorylation of transcription factor CREB activating the CREB signaling pathway, pCREB recruits more CBP, and p300 initiates the transcriptional machinery, including histone acetyltransferase. Alternatively, calcium or other ELFEF-activated signals could induce histone modifications and chromatin unravelling, leading to the pCREB binding and the start of transcription. On the other hand, the pCREB is able to bind to the promoter of a series of miRNAs to modulate their expression. In addition, CREB itself as well as the epigenetics mechanisms could affect the expression of BDNF which plays a critical role in the activities of NSCs.

Overall, these mechanisms discussed above have the potential to mediate rTMS effects on NSCs (Figure 2). However, there might be some enigmatic affiliation among them, suggesting that further investigations should explore the underlying interactions among these mechanisms in order to fully understand the rTMS effects on NSCs, thereby elucidating the additional pathways involved.

## 6. Clinical Applications

### 6.1. Overview of the Current Applications

The growing interest in noninvasive brain stimulation generated by TMS has led to its widespread application in various neurological and psychiatric disorders and to rehabilitation applications for better diagnostic and therapeutic purposes. rTMS has been used for diagnostics and treatments of refractory brain diseases, including depression, Parkinson's disease, multiple sclerosis, dementia, stroke, auditory hallucination, neural tinnitus, anxiety, sleep disorder, obsessive-compulsive disorder, epilepsy, schizophrenia, PSTD, substance addiction, and so forth. In particular, rTMS is a treatment used worldwide, with definite therapeutic effects for depression patients resistant to antidepressant medications, and it was authorized for use in treatment-resistant depression by the US Food and Drug Administration (FDA) in 2008. In addition, Alzheimer's disease (AD) is a widespread degenerative disease whose early diagnosis and prevention is critically important. The diagnosis of early AD has been achieved using TMS coupled with peripheral magnetic stimulation [22]. Additionally, the effective treatment of the cognitive problems and language deficits caused by AD has been realized using low- and high-frequency repetitive magnetic stimulation (MS) [9, 12]. Currently, more and more researchers are actively studying rTMS in the clinic with the hope of using it as a promising treatment for several psychiatric and neurological disorders [18, 23].

### 6.2. Opportunities and Challenges of EMF-Based NSC Therapy

As discussed above, EMF is widely used in different types of diseases due to the diverse effects it exerts on the human body. The interaction between EMF and NSCs were the focus of this article. EMF's positive effects on the natural properties of NSCs, including the ability to self-renew and multidifferentiate, suggests that stem cell therapy is a powerful method to treat refractory diseases. To some degree, it may be the ultimate weapon to fight these diseases. Therefore, we propose that EMF-based NSCs are the future of stem cell therapy and should be the focus of research on the development of biological science technology. However, their use could face barriers such as ethical considerations, the isolation of the stem cells, and the safety or stability of their application. In contrast, the application of EMF, as a noninvasive technique, could easily and remarkably influence NSCs, particularly endogenous NSCs, thereby providing a new way to solve the current issues associated with the use of stem cells as a therapy for neurological diseases. Opportunities come with challenges; for example, (1) a broad range of parameters requires additional clinical tests and animal experiments. (2) The safety of EMF-based NSC therapy is unclear, and there is no doubt that high-intensity EMF can cause damage to cells and may induce the mutation of the cells. (3) The relationship between the biological parameters and the physical parameters should be elaborated in the future.

## 7. Limitations

rTMS has a definite positive effect on the brain and has been widely exploited in the clinic. However, rTMS is characterized by certain limitations that restrict the use of the technique and its applications. (1) Lack of focus: it is difficult to stimulate precise regions. (2) As an interdisciplinary field requiring extensive knowledge of physiology, its mechanisms are complicated and remain elusive. We lack the requisite understanding of how rTMS regulates biological processes. Therefore, in the future, researchers from different fields are encouraged to cooperate with one another and to combine their studies for a better understanding of the underlying mechanisms. (3) There is essentially little nonhuman experimental data demonstrating how TMS works at the cellular and molecular levels (Table 1) [2]. Thus, a better understanding of rTMS-induced neural plasticity is needed to optimize treatment protocols and to develop new diagnostic and therapeutic strategies using rTMS [3]. Thus, there is an enormous parameter space to explore by conducting appropriate experiments and clinical practices, carefully recording the data, which will provide novel insight into the dose, orientation, frequency, intensity, period, and so forth, offering numerous diverse possible applications. (4) Because of the high voltage and strong currents, the safety of TMS needs further discussion. It is generally recognized that single-pulsed TMS is safe, while high-frequency or high-intensity TMS may cause unexpected side effects, which means that unified clinical guidelines and more tests are required.

## 8. Perspective and Conclusion

Despite its limitations, TMS is a promising therapeutic tool for many refractory neural diseases. It is noninvasive and has a clear positive influence on different parts of the brain, especially on NSCs. Nevertheless, NSCs are promising for traumatic, degenerative, and psychiatric diseases. All these findings contributed to TMS being deemed as a brain science technology of the 21st century. In the near future, we should perfect the technique of TMS, and more in-depth studies should be performed. Clinical applications must be expanded to collect more data regarding the modality.

All the assumptions made in this review are based on previously reported studies, although there are many discrepancies among reported results. However, we must mention that different research circumstances, for instance, will help guide us toward a more detailed understanding of rTMS. We believe that the efforts of excellent researchers will accelerate the development of TMS applications, making it a powerful tool to treat people who are surviving with painful diseases.

## Figures and Tables

**Table 1:** Current studies of electromagnetic fields and transcranial magnetic stimulation.

Category	Model	Method of stimulation	Field intensity	Stimulation pattern	Stimulation duration	Post stim assessment	Region assessed	Main effect of TMS	Potential mechanism	Reference
In vitro	Embryonic neural stem cells (eNSCs) from embryonic day 13.5 (E13.5) BALB/c mice	sXc-ELF exposure system (IT'IS Foundation, Zurich, Switzerland)	1 mT	Exposed to ELF-EMF (50 Hz, 1 mT) for 1, 2, and 3 days with 4 hours per day	1/2/3 days	Same day	eNSCs in vitro	The neuronal differentiation ↑ Neurite outgrowth ↑	The expression of TRPC1 and proneural genes (NeuroD and Ngn1) ↑	Ma et al. (2016) [95]
	Embryonic neural stem cells (eNSCs) from embryonic day 13.5 (E13.5) BALB/c mice	sXc-ELF exposure system (IT'IS Foundation, Zurich, Switzerland)	0.5 mT, 1 mT, and 2 mT	At a frequency of 50 Hz sinusoidal waves with magnetic intensities of 0.5 mT, 1 mT, and 2 mT for 3 days or with a magnetic intensity of 2 mT for 1 day, 2 days, and 3 days, with an intermittent cycle of 5 min on/10 min	1/2/3 days	Same day	eNSCs in vitro	Intermittent exposure to ELF-EMF no change in proliferation of eNSCs and percentages of Tuj1-positive cells and GFAP	Sox2↓ Math1, Math3, Ngn1 Tuj1 mRNA levels ↑	Ma et al. (2014) [96]
	NSCs from the hippocampus of neonatal 3-day-old SD rats	rTMS 90 mm figure-of-eight coil (Yirui De, CCY-I, Wuhan, China)	50% of the device's maximum power (peak value 3.5 T)	200/400/600/800/1000 pulses per day, 10 s trains with 10 Hz frequency	3 days	Same day	NSCs in vitro	NSC proliferation in vitro in a dose-dependent manner ↑	miR-106b expression ↑ miR-106b/p21/cdks/cyclin pathway	Liu et al. (2015) [65]
	Neonatal rat neural stem cells	HMF-S20-type pulsed magnetic field device (manufactured by High Magnetic Center of Huazhong University of Science and Technology)	0.5–10 T	0.1 Hz, 0.5–10 Tesla (T) [8 groups of B–I, resp.]	5 stimuli of high-intensity pulsed electromagnetic field (HIPEMF)	24th h, 48th h, 72nd h, and 7th day	NSCs in vitro	6.0–10.0 T peak intensity led to poor growth of rat NSCs in vitro, and in the condition of 0.5–4.0 T peak intensity, HIPEMF stimulated the proliferation of rat NSCs	___	Meng et al. (2009) [52]
	NSCs from postnatal day 0 (P0) CD-1 mice	Solenoid generating alternating EFs	1 mT	50 Hz continuously	Up to 12 days	Same day	NSCs in vitro	Neurogenesis ↑	Cav1 channel activity ↑ intracellular Ca2þ signaling ↑	Piacentini et al. (2008) [73]
	Normal, healthy mice	rTMS (model 9000 MS, Neurosoft, Ivanovo, Russia) 100 mm circular coil	Maximum output intensity of the device	1 Hz and 30 Hz	1 Hz: received 150 pulses/day (5 second train, 10 second pause) in 450 seconds and 30 Hz group received 150 pulses/day (1 second train, 5 second pause) in 30 seconds. 7 or 14 consecutive days	7 days or 14 days	NSCs from subventricular zone	NS/PC proliferation and neuronal differentiation ↑	BDNF; activation of different neurotransmitter system	Abbasnia et al. (2015) [16]

In vivo	NSCs from the hippocampi of newborn C57bl/6 mice	Solenoid generating alternating EFs characterized by a sinusoidal waveform with amplitudes of 5–1000 *μ*T and frequencies of 1–100 Hz	1 mT	50 Hz; 3.5 h/day	12 days (12 D × 3.5 h)	One month	Hippocampal dentate gyrus	Proliferation and neuronal differentiation of NSCs ↑	pCREB signaling pathway epigenetic modulation	Leone et al. (2014) [68]
	Adult male SD rats model of depression in chronic unpredictable stress	rTMS round coil (inner diameter, 2.5 cm; outer diameter, 5 cm; custom-made YIRD, China)	1.26 T	15 Hz, 15 s trains, 900 pulses daily	7 days	1 day	Hippocampal dentate gyrus	NSPC proliferation ↑	BDNF expression; BDNF/ERK signaling pathway ↑	Chen et al. (2015) [57]
	Ischemic injury in rats	rTMS round prototype coil 6 cm in diameter with 3.5 T peak magnetic welds (YRD-CCI, Wuhan, China)	Stimulation intensity was set at 120% of the average resting motor threshold (RMT), namely, 26% of the maximum output of the stimulator	Stimulation for 3 s followed by rest for 50 s, which was repeated ten times (300 pulses per day) at the rate of 10 Hz	7 days	12 h	The ischemic cortex subventricular zone (SVZ)	Adult NSC proliferation ↑	miR-25 expression ↑ miR-25/p57/cdks/cyclins pathway	Guo et al. (2014) [55]
	Ischemic injury in male Wistar rats	rTMS figure-of-eight coil (CCY-II, Wuhan Yiruide Medical Equipment, Wuhan, China)	20 Hz group: 120% RMT (24% of the maximum stimulator output) iTBS80% RMT (16% of the maximum stimulator output)	40 trains at 20 Hz for 1 s 15 s interval 20 trains with 600 pulses	10 days	2 days	Ipsilateral SVZ peri-infarct striatum	Improvements of functional recovery ↑ Volume of the infarct area ↓ Migration, differentiation, and proliferation ↑	BDNF/TrkB signaling pathway; ↑ expression levels of BDNF ↑	Luo et al. (2017) [51]
	Normal, healthy mice	Deep brain magnetic stimulation via two coils placed either side of the cage	10 mT peak	Varying pulsed magnetic fields	4 or 7 days	1 day	Dentate gyrus	NSPC proliferation Neurogenesis ↑	c-fos gene; ↑ expression level of fgf1b ↑; stimulate neural activity in certain brain regions by modulating the balance between excitatory and inhibitory neurons	Zhang et al. (2014) [97]
	Nigrostriatal lesion and chromaffin cell transplant in rats	Oscillatory magnetic field via two 7 cm Hemholtz coils positioned dorsal and ventral to the head	0.7 mT	60 Hz, 2 h morning and afternoon	60 Hz, 2 h morning and afternoon	Same day	SVZ	NSPC proliferation ↑	—	Arias-Carrion et al. (2004) [50]
	Normal, healthy rats	rTMS figure-of-eight coil	70% maximum power	25 Hz, 4 × 10 s trains daily	14 days	1 day	Dentate gyrus	NSPC proliferation neurogenesis ↑	—	Ueyama et al. (2011) [53]
	Normal, healthy C57 mice	Solenoid generating alternating EFs	1 mT	1 to 7 h/day for 7 days	7 days	Same day	Dentate gyrus (DG)	NSC proliferation ↑	Expression of Mash1, Hes1 ↑, and NeuroD2 mRNAs; ↑ Ca^2+^ influx ↑	Cuccurazzu et al. (2010) [49]

## References

[1] Barker A., Freeston I., Jalinous R., Merton P., Morton H. (1985). Magnetic stimulation of the human brain. *Journal of Physiology-London*.

[2] Dayan E., Censor N., Buch E. R., Sandrini M., Cohen L. G. (2013). Noninvasive brain stimulation: from physiology to network dynamics and back. *Nature Neuroscience*.

[3] Parkin B. L., Ekhtiari H., Walsh V. F. (2015). Non-invasive human brain stimulation in cognitive neuroscience: a primer. *Neuron*.

[4] Yozbatiran N., Alonso-Alonso M., See J. (2009). Safety and behavioral effects of high-frequency repetitive transcranial magnetic stimulation in stroke. *Stroke*.

[5] Houdayer E., Degardin A., Cassim F., Bocquillon P., Derambure P., Devanne H. (2008). The effects of low- and high-frequency repetitive TMS on the input/output properties of the human corticospinal pathway. *Experimental Brain Research*.

[6] Clarke D. L., Johansson C. B., Wilbertz J. (2000). Generalized potential of adult neural stem cells. *Science*.

[7] Anderson C. M., Swanson R. A. (2000). Astrocyte glutamate transport: review of properties, regulation, and physiological functions. *Glia*.

[8] Minguell J. J., Erices A., Conget P. (2001). Mesenchymal stem cells. *Experimental Biology and Medicine*.

[9] Kobilo T., Yuan C., van Praag H. (2011). Endurance factors improve hippocampal neurogenesis and spatial memory in mice. *Learning & Memory*.

[10] Podda M. V., Leone L., Barbati S. A. (2014). Extremely low-frequency electromagnetic fields enhance the survival of newborn neurons in the mouse hippocampus. *The European Journal of Neuroscience*.

[11] Lin R., Iacovitti L. (2015). Classic and novel stem cell niches in brain homeostasis and repair. *Brain Research*.

[12] Hayrapetyan A., Jansen J. A., van den Beucken J. J. (2015). Signaling pathways involved in osteogenesis and their application for bone regenerative medicine. *Tissue Engineering Part B, Reviews*.

[13] Gage F. H. (2000). Mammalian neural stem cells. *Science*.

[14] Ming G. L., Song H. (2011). Adult neurogenesis in the mammalian brain: significant answers and significant questions. *Neuron*.

[15] Kempermann G. (2015). Activity dependency and aging in the regulation of adult neurogenesis. *Cold Spring Harbor Perspectives in Biology*.

[16] Abbasnia K., Ghanbari A., Abedian M., Ghanbari A., Sharififar S., Azari H. (2015). The effects of repetitive transcranial magnetic stimulation on proliferation and differentiation of neural stem cells. *Anatomy & Cell Biology*.

[17] Liu R. H., Morassutti D. J., Whittemore S. R., Sosnowski J. S., Magnuson D. S. (1999). Electrophysiological properties of mitogen-expanded adult rat spinal cord and subventricular zone neural precursor cells. *Experimental Neurology*.

[18] Doetsch F., Garcia-Verdugo J. M., Alvarez-Buylla A. (1997). Cellular composition and three-dimensional organization of the subventricular germinal zone in the adult mammalian brain. *The Journal of Neuroscience: The Official Journal of the Society for Neuroscience*.

[19] Fukuda S., Kato F., Tozuka Y., Yamaguchi M., Miyamoto Y., Hisatsune T. (2003). Two distinct subpopulations of nestin-positive cells in adult mouse dentate gyrus. *The Journal of Neuroscience: The Official Journal of the Society for Neuroscience*.

[20] Cesetti T., Obernier K., Bengtson C. P. (2009). Analysis of stem cell lineage progression in the neonatal subventricular zone identifies EGFR+/NG2- cells as transit-amplifying precursors. *Stem Cells*.

[21] Smith D. O., Rosenheimer J. L., Kalil R. E. (2008). Delayed rectifier and A-type potassium channels associated with Kv 2.1 and Kv 4.3 expression in embryonic rat neural progenitor cells. *PLoS One*.

[22] Owens D. F., Boyce L. H., Davis M. B., Kriegstein A. R. (1996). Excitatory GABA responses in embryonic and neonatal cortical slices demonstrated by gramicidin perforated-patch recordings and calcium imaging. *The Journal of Neuroscience: The Official Journal of the Society for Neuroscience*.

[23] Cai J., Cheng A., Luo Y. (2004). Membrane properties of rat embryonic multipotent neural stem cells. *Journal of Neurochemistry*.

[24] Nguyen L., Malgrange B., Belachew S. (2002). Functional glycine receptors are expressed by postnatal nestin-positive neural stem/progenitor cells. *The European Journal of Neuroscience*.

[25] Verkhratsky A., Steinhauser C. (2000). Ion channels in glial cells. *Brain Research Reviews*.

[26] Scheffler B., Walton N. M., Lin D. D. (2005). Phenotypic and functional characterization of adult brain neuropoiesis. *Proceedings of the National Academy of Sciences of the United States of America*.

[27] Yasuda T., Bartlett P. F., Adams D. J. (2008). K(ir) and K(v) channels regulate electrical properties and proliferation of adult neural precursor cells. *Molecular and Cellular Neurosciences*.

[28] Piper D. R., Mujtaba T., Rao M. S., Lucero M. T. (2000). Immunocytochemical and physiological characterization of a population of cultured human neural precursors. *Journal of Neurophysiology*.

[29] Schaarschmidt G., Wegner F., Schwarz S. C., Schmidt H., Schwarz J. (2009). Characterization of voltage-gated potassium channels in human neural progenitor cells. *PLoS One*.

[30] Feldman D. H., Thinschmidt J. S., Peel A. L., Papke R. L., Reier P. J. (1996). Differentiation of ionic currents in CNS progenitor cells: dependence upon substrate attachment and epidermal growth factor. *Experimental Neurology*.

[31] Dubois J. M., Rouzaire-Dubois B. (1993). Role of potassium channels in mitogenesis. *Progress in Biophysics and Molecular Biology*.

[32] Pardo L. A. (2004). Voltage-gated potassium channels in cell proliferation. *Physiology*.

[33] Kunzelmann K. (2005). Ion channels and cancer. *The Journal of Membrane Biology*.

[34] Lang F., Foller M., Lang K. S. (2005). Ion channels in cell proliferation and apoptotic cell death. *The Journal of Membrane Biology*.

[35] Liebau S., Vaida B., Proepper C. (2007). Formation of cellular projections in neural progenitor cells depends on SK3 channel activity. *Journal of Neurochemistry*.

[36] Spitzer N. C. (2006). Electrical activity in early neuronal development. *Nature*.

[37] Kong H., Fan Y., Xie J. (2008). AQP4 knockout impairs proliferation, migration and neuronal differentiation of adult neural stem cells. *Journal of Cell Science*.

[38] Luo C. X., Zhu X. J., Zhang A. X. (2005). Blockade of L-type voltage-gated Ca channel inhibits ischemia-induced neurogenesis by down-regulating iNOS expression in adult mouse. *Journal of Neurochemistry*.

[39] Fiorio Pla A., Maric D., Brazer S. C. (2005). Canonical transient receptor potential 1 plays a role in basic fibroblast growth factor (bFGF)/FGF receptor-1-induced Ca2+ entry and embryonic rat neural stem cell proliferation. *The Journal of Neuroscience: The Official Journal of the Society for Neuroscience*.

[40] Rossi S., Hallett M., Rossini P. M., Pascual-Leone A., Safety of TMS Consensus Group (2009). Safety, ethical considerations, and application guidelines for the use of transcranial magnetic stimulation in clinical practice and research. *Clinical Neurophysiology: Official Journal of the International Federation of Clinical Neurophysiology*.

[41] Bilek E., Schafer A., Ochs E. (2013). Application of high-frequency repetitive transcranial magnetic stimulation to the DLPFC alters human prefrontal-hippocampal functional interaction. *The Journal of Neuroscience: The Official Journal of the Society for Neuroscience*.

[42] Lisanby S. H., Datto C. J., Szuba M. P. (2000). ECT and TMS: past, present, and future. *Depression and Anxiety*.

[43] Touge T., Gerschlager W., Brown P., Rothwell J. C. (2001). Are the after-effects of low-frequency rTMS on motor cortex excitability due to changes in the efficacy of cortical synapses?. *Clinical Neurophysiology: Official Journal of the International Federation of Clinical Neurophysiology*.

[44] Francis F., Koulakoff A., Boucher D. (1999). Doublecortin is a developmentally regulated, microtubule-associated protein expressed in migrating and differentiating neurons. *Neuron*.

[45] Gleeson J. G., Lin P. T., Flanagan L. A., Walsh C. A. (1999). Doublecortin is a microtubule-associated protein and is expressed widely by migrating neurons. *Neuron*.

[46] Nacher J., Crespo C., McEwen B. S. (2001). Doublecortin expression in the adult rat telencephalon. *The European Journal of Neuroscience*.

[47] Martin L. A., Tan S. S., Goldowitz D. (2002). Clonal architecture of the mouse hippocampus. *The Journal of Neuroscience: The Official Journal of the Society for Neuroscience*.

[48] Sherafat M. A., Heibatollahi M., Mongabadi S., Moradi F., Javan M., Ahmadiani A. (2012). Electromagnetic field stimulation potentiates endogenous myelin repair by recruiting subventricular neural stem cells in an experimental model of white matter demyelination. *Journal of Molecular Neuroscience*.

[49] Cuccurazzu B., Leone L., Podda M. V. (2010). Exposure to extremely low-frequency (50 Hz) electromagnetic fields enhances adult hippocampal neurogenesis in C57BL/6 mice. *Experimental Neurology*.

[50] Arias-Carrion O., Verdugo-Diaz L., Feria-Velasco A. (2004). Neurogenesis in the subventricular zone following transcranial magnetic field stimulation and nigrostriatal lesions. *Journal of Neuroscience Research*.

[51] Luo J., Zheng H., Zhang L. (2017). High-frequency repetitive transcranial magnetic stimulation (rTMS) improves functional recovery by enhancing neurogenesis and activating BDNF/TrkB signaling in ischemic rats. *International Journal of Molecular Sciences*.

[52] Meng D., Xu T., Guo F., Yin W., Peng T. (2009). The effects of high-intensity pulsed electromagnetic field on proliferation and differentiation of neural stem cells of neonatal rats *in vitro*. *Journal of Huazhong University of Science and Technology Medical Sciences = Hua zhong ke ji da xue xue bao Yi xue Ying De wen ban = Huazhong keji daxue xuebao Yixue Yingdewen ban*.

[53] Ueyama E., Ukai S., Ogawa A. (2011). Chronic repetitive transcranial magnetic stimulation increases hippocampal neurogenesis in rats. *Psychiatry and Clinical Neurosciences*.

[54] Feng S. F., Shi T. Y., Fan Y., Wang W. N., Chen Y. C., Tan Q. R. (2012). Long-lasting effects of chronic rTMS to treat chronic rodent model of depression. *Behavioural Brain Research*.

[55] Guo F., Han X., Zhang J. (2014). Repetitive transcranial magnetic stimulation promotes neural stem cell proliferation via the regulation of MiR-25 in a rat model of focal cerebral ischemia. *PLoS One*.

[56] Muller M. B., Toschi N., Kresse A. E., Post A., Keck M. E. (2000). Long-term repetitive transcranial magnetic stimulation increases the expression of brain-derived neurotrophic factor and cholecystokinin mRNA, but not neuropeptide tyrosine mRNA in specific areas of rat brain. *Neuropsychopharmacology: Official Publication of the American College of Neuropsychopharmacology*.

[57] Chen Y. H., Zhang R. G., Xue F. (2015). Quetiapine and repetitive transcranial magnetic stimulation ameliorate depression-like behaviors and up-regulate the proliferation of hippocampal-derived neural stem cells in a rat model of depression: the involvement of the BDNF/ERK signal pathway. *Pharmacology, Biochemistry, and Behavior*.

[58] Zhao C., Sun G., Li S., Shi Y. (2009). A feedback regulatory loop involving microRNA-9 and nuclear receptor TLX in neural stem cell fate determination. *Nature Structural & Molecular Biology*.

[59] Cremisi F. (2013). MicroRNAs and cell fate in cortical and retinal development. *Frontiers in Cellular Neuroscience*.

[60] Perruisseau-Carrier C., Jurga M., Forraz N., McGuckin C. P. (2011). miRNAs stem cell reprogramming for neuronal induction and differentiation. *Molecular Neurobiology*.

[61] Brett J. O., Renault V. M., Rafalski V. A., Webb A. E., Brunet A. (2011). The microRNA cluster miR-106b~25 regulates adult neural stem/progenitor cell proliferation and neuronal differentiation. *Aging*.

[62] Peck B., Schulze A. (2011). A role for the cancer-associated miR-106b~25 cluster in neuronal stem cells. *Aging*.

[63] Poliseno L., Salmena L., Riccardi L. (2010). Identification of the miR-106b~25 microRNA cluster as a proto-oncogenic PTEN-targeting intron that cooperates with its host gene MCM7 in transformation. *Science Signaling*.

[64] Zhao Z. N., Bai J. X., Zhou Q. (2012). TSA suppresses miR-106b-93-25 cluster expression through downregulation of MYC and inhibits proliferation and induces apoptosis in human EMC. *PLoS One*.

[65] Liu H., Han X. H., Chen H., Zheng C. X., Yang Y., Huang X. L. (2015). Repetitive magnetic stimulation promotes neural stem cells proliferation by upregulating MiR-106b *in vitro*. *Journal of Huazhong University of Science and Technology Medical sciences = Hua zhong ke ji da xue xue bao Yi xue Ying De wen ban = Huazhong keji daxue xuebao Yixue Yingdewen ban*.

[66] Luo J., Hu X., Zhang L. (2014). Physical exercise regulates neural stem cells proliferation and migration via SDF-1alpha/CXCR4 pathway in rats after ischemic stroke. *Neuroscience Letters*.

[67] Morris D. C., Chopp M., Zhang L., Lu M., Zhang Z. G. (2010). Thymosin beta4 improves functional neurological outcome in a rat model of embolic stroke. *Neuroscience*.

[68] Leone L., Fusco S., Mastrodonato A. (2014). Epigenetic modulation of adult hippocampal neurogenesis by extremely low-frequency electromagnetic fields. *Molecular Neurobiology*.

[69] Bai G., Sheng N., Xie Z. (2007). Id sustains Hes1 expression to inhibit precocious neurogenesis by releasing negative autoregulation of Hes1. *Developmental Cell*.

[70] Hatakeyama J., Kageyama R. (2004). Retinal cell fate determination and bHLH factors. *Seminars in Cell & Developmental Biology*.

[71] Ishibashi M., Ang S. L., Shiota K., Nakanishi S., Kageyama R., Guillemot F. (1995). Targeted disruption of mammalian hairy and enhancer of split homolog-1 (HES-1) leads to up-regulation of neural helix-loop-helix factors, premature neurogenesis, and severe neural tube defects. *Genes & Development*.

[72] Tomita K., Nakanishi S., Guillemot F., Kageyama R. (1996). Mash1 promotes neuronal differentiation in the retina. *Genes to Cells: Devoted to Molecular & Cellular Mechanisms*.

[73] Piacentini R., Ripoli C., Mezzogori D., Azzena G. B., Grassi C. (2008). Extremely low-frequency electromagnetic fields promote *in vitro* neurogenesis via upregulation of Ca(v)1-channel activity. *Journal of Cellular Physiology*.

[74] Grassi C., D'Ascenzo M., Torsello A. (2004). Effects of 50 Hz electromagnetic fields on voltage-gated Ca2+ channels and their role in modulation of neuroendocrine cell proliferation and death. *Cell Calcium*.

[75] D'Ascenzo M., Piacentini R., Casalbore P. (2006). Role of L-type Ca2+ channels in neural stem/progenitor cell differentiation. *The European Journal of Neuroscience*.

[76] Sinnegger-Brauns M. J., Huber I. G., Koschak A. (2009). Expression and 1,4-dihydropyridine-binding properties of brain L-type calcium channel isoforms. *Molecular Pharmacology*.

[77] Ince-Dunn G., Hall B. J., Hu S. C. (2006). Regulation of thalamocortical patterning and synaptic maturation by NeuroD2. *Neuron*.

[78] Deisseroth K., Singla S., Toda H., Monje M., Palmer T. D., Malenka R. C. (2004). Excitation-neurogenesis coupling in adult neural stem/progenitor cells. *Neuron*.

[79] Ramirez M., Hernandez-Montoya J., Sanchez-Serrano S. L. (2012). GABA-mediated induction of early neuronal markers expression in postnatal rat progenitor cells in culture. *Neuroscience*.

[80] West A. E., Chen W. G., Dalva M. B. (2001). Calcium regulation of neuronal gene expression. *Proceedings of the National Academy of Sciences of the United States of America*.

[81] Jagasia R., Steib K., Englberger E. (2009). GABA-cAMP response element-binding protein signaling regulates maturation and survival of newly generated neurons in the adult hippocampus. *The Journal of Neuroscience: The Official Journal of the Society for Neuroscience*.

[82] Merz K., Herold S., Lie D. C. (2011). CREB in adult neurogenesis—master and partner in the development of adult-born neurons?. *The European Journal of Neuroscience*.

[83] Bito H., Takemoto-Kimura S. (2003). Ca(2+)/CREB/CBP-dependent gene regulation: a shared mechanism critical in long-term synaptic plasticity and neuronal survival. *Cell Calcium*.

[84] Wang J., Weaver I. C., Gauthier-Fisher A. (2010). CBP histone acetyltransferase activity regulates embryonic neural differentiation in the normal and Rubinstein-Taybi syndrome brain. *Developmental Cell*.

[85] Chatterjee S., Mizar P., Cassel R. (2013). A novel activator of CBP/p300 acetyltransferases promotes neurogenesis and extends memory duration in adult mice. *The Journal of Neuroscience: The Official Journal of the Society for Neuroscience*.

[86] Le Q., Qu Y., Tao Y., Zhu S. (2014). Effects of repetitive transcranial magnetic stimulation on hand function recovery and excitability of the motor cortex after stroke: a meta-analysis. *American Journal of Physical Medicine & Rehabilitation*.

[87] Yoon K. J., Lee Y. T., Han T. R. (2011). Mechanism of functional recovery after repetitive transcranial magnetic stimulation (rTMS) in the subacute cerebral ischemic rat model: neural plasticity or anti-apoptosis?. *Experimental Brain Research*.

[88] Barreto G., White R. E., Ouyang Y., Xu L., Giffard R. G. (2011). Astrocytes: targets for neuroprotection in stroke. *Central Nervous System Agents in Medicinal Chemistry*.

[89] Liu Z., Chopp M. (2016). Astrocytes, therapeutic targets for neuroprotection and neurorestoration in ischemic stroke. *Progress in Neurobiology*.

[90] Bagley J. A., Belluscio L. (2010). Dynamic imaging reveals that brain-derived neurotrophic factor can independently regulate motility and direction of neuroblasts within the rostral migratory stream. *Neuroscience*.

[91] Schabitz W. R., Steigleder T., Cooper-Kuhn C. M. (2007). Intravenous brain-derived neurotrophic factor enhances poststroke sensorimotor recovery and stimulates neurogenesis. *Stroke*.

[92] Chiaramello S., Dalmasso G., Bezin L. (2007). BDNF/TrkB interaction regulates migration of SVZ precursor cells via PI3-K and MAP-K signalling pathways. *The European Journal of Neuroscience*.

[93] Young C. C., Brooks K. J., Buchan A. M., Szele F. G. (2011). Cellular and molecular determinants of stroke-induced changes in subventricular zone cell migration. *Antioxidants & Redox Signaling*.

[94] Lee Y. S., Chio C. C., Chang C. P. (2013). Long course hyperbaric oxygen stimulates neurogenesis and attenuates inflammation after ischemic stroke. *Mediators of Inflammation*.

[95] Ma Q., Chen C., Deng P. (2016). Extremely low-frequency electromagnetic fields promote in vitro neuronal differentiation and neurite outgrowth of embryonic neural stem cells via up-regulating TRPC1. *PLoS One*.

[96] Ma Q., Deng P., Zhu G. (2014). Extremely low-frequency electromagnetic fields affect transcript levels of neuronal differentiation-related genes in embryonic neural stem cells. *PLoS One*.

[97] Zhang Y., Mao R. R., Chen Z. F. (2014). Deep-brain magnetic stimulation promotes adult hippocampal neurogenesis and alleviates stress-related behaviors in mouse models for neuropsychiatric disorders. *Molecular Brain*.

